# *In Vitro* Assessment of Yeasts Strains with Probiotic Attributes for Aquaculture Use

**DOI:** 10.3390/foods12010124

**Published:** 2022-12-26

**Authors:** Camelia Filofteia Diguță, Constanța Mihai, Radu Cristian Toma, Carmen Cîmpeanu, Florentina Matei

**Affiliations:** 1 Faculty of Biotechnologies, University of Agronomic Sciences and Veterinary Medicine of Bucharest, 59, Mărăști Blvd., District 1, 011464 Bucharest, Romania; 2 Faculty of Land Reclamation and Environmental Engineering, University of Agronomic Sciences and Veterinary Medicine of Bucharest, 59, Mărăști Blvd., District 1, 011464 Bucharest, Romania

**Keywords:** *Saccharomyces*, non-*Saccharomyces*, probiotic properties, safety issue

## Abstract

This study aimed to investigate *in vitro* the probiotic potential of three yeasts strains (BB06, OBT05, and MT07) isolated from agro-food natural sources. Screening was performed, including several functional, technological, and safety aspects of the yeast strains, in comparison to a reference *Saccharomyces boulardii*, to identify the ones with suitable probiotic attributes in aquaculture. The yeast strains were identified by 5.8S rDNA-ITS region sequencing as *Metschnikowia pulcherrima* OBT05, *Saccharomyces cerevisiae* BB06, and *Torulaspora delbrueckii* MT07. All yeast strains were tolerant to different temperatures, sodium chloride concentrations, and wide pH ranges. *S. cerevisiae* BB06 showed a strong and broad antagonistic activity. Moreover, the *S. cerevisiae* strain exhibited a high auto-aggregation ability (92.08 ± 1.49%) and good surface hydrophobicity to hexane as a solvent (53.43%). All of the yeast strains have excellent antioxidant properties (>55%). The high survival rate in the gastrointestinal tract (GIT) can promote yeast isolates as probiotics. All yeast strains presented a resistance pattern to the antibacterial antibiotics. Non-hemolytic activity was detected. Furthermore, freeze-drying with cryoprotective agents maintained a high survival rate of yeast strains, in the range of 74.95–97.85%. According to the results obtained, the *S. cerevisiae* BB06 strain was found to have valuable probiotic traits.

## 1. Introduction

Fish production in farm aquaculture has increased significantly in the past years. Nevertheless, semi-intensive or intensive production systems have exposed the fish to prolonged stressful conditions with a negative impact on their well-being by suppressing their immunity, thereby increasing the fish’s susceptibility to pathogens, all of which can translate into low performance [1,2,3]. However, the uncontrolled and prolonged application of antibacterial antibiotics to treat bacterial diseases that occur most frequently in the fish leads to significant changes in the microflora of the fish, their accumulation in the tissues [4,5], a decrease in immunity, and the appearance of antibiotic-resistant bacteria, which represent a serious threat [6,7,8,9]. The consumption of contaminated fish products could have a negative impact on human health [10,11]. Currently, the use of probiotics has been promoted as a viable, safe alternative method for sustainable aquaculture [5,12,13,14,15,16,17,18,19]. Probiotics are viable microorganisms which, when administered in adequate amounts, provide health benefits to the host [20,21]. Lactic bacteria and *Bacillus* spp. have been the most researched probiotics and widely administered in aquaculture via diet or water, either by themselves, as a mix of different species, or combined with other ingredients such as prebiotics to improve performance and health status of fishes [14,19,22,23,24,25,26,27]. However, the possible risk to transfer antibiotic resistance genes through bacterial probiotics to pathogenic bacteria is a threat [28,29]. Recently, yeasts have gained interest as promising probiotics to improve fish health [30,31,32,33,34,35,36]. The biotechnological importance of yeasts is well-known by their use as starter cultures in the production of high-value functional food with health benefits [37,38]. Moreover, yeasts have been proposed as an alternative for replacing fish meal, being considered a complex protein source in aquafeeds [39,40]. One of the main advantages of yeasts as probiotics supplements is the resistance to antibacterial antibiotics, which makes them suitable for use during antibiotic treatment. *Saccharomyces cerevisiae* and *S.cerevisiae* var. *boulardii* (or *S. boulardii*) isolated from a wide range of sources are extensively studied yeast species as probiotics [41,42,43,44]. *S. cerevisiae* var. *boulardii* was recognized as safe (receiving the Qualified Presumption of Safety ”QPS” status) by the European Food Safety Authority (EFSA) and the only market is as probiotic products [45]. Moreover, scientific studies have increased and focused on the isolation and characterization of the new non-*Saccharomyces* probiotic species (e.g., *Debaryomyces hansenii, Kluyveromyces lactis, K. marxianus, Torulaspora delbrueckii, Pichia fermentas, P. guilliermondii*, *P. kudriavzevi,* and *Yarrowia lipolytica*) [46,47,48,49,50,51,52]. In this regard, several review articles describing different selection criteria of probiotics have been published in the past several years [53,54,55,56,57,58].

Several researchers have observed that the administration of yeast probiotics supplements in fish feed improved growth rate [33,34,35], feed digestion [30,31,36,58], stress tolerance [33], the immune system [31,36], and disease control [30,32,33,35]. 

The main objective of this study was the assessment of three *Saccharomyces* and non-*Saccharomyces* yeast strains (BB06, OBT05, and MT07) by *in vitro* testing of their functional and safety properties, as promising probiotic candidates in fish feed.

## 2. Materials and Methods

### 2.1. Yeast Strains and Culture Conditions

In this study, a BB06 strain (isolated from grapes)*,* OBT05 strain (isolated from barley)*,* and MT07 strain (isolated from grapes), belonging to the Microorganisms Collection of UASMVB (Bucharest, Romania) were used. A commercial probiotic yeast, *S. cerevisiae* var. *boulardii*, was used as an indicator strain. All strains were stored at −20 °C in YPD broth (Scharlau, Barcelona, Spain) containing glycerol (30% *v*/*v*); initially, yeast strains were reactivated in YEPD Agar (Scharlau, Barcelona, Spain) at 26 °C for 24 h.

### 2.2. Yeasts Identification

Yeast strains were identified according to 5.8S-ITS gene sequencing. Briefly, yeast strains were grown in YEPD broth overnight, and cells were recovered by centrifugation at 5000× *g* for 10 min. The extraction of fungal DNA was performed using a ZR Fungal/Bacterial DNA kit (Zymo Research, Irvine, CA, USA), and finally quantified with SpectraMax^®^ QuickDrop™ (Molecular Devices, San Jose, CA, USA). PCR amplification of the 5.8S-ITS gene was performed in 50 μL of 10× DreamTaq Green Buffer (including 20 mmol MgCl_2_), 0.2 mM of dNTPs, 0.5 µM forward ITS1 primer (5′-TCCGTAGGTGAACCTGCGG-3′), 0.5 µM reverse ITS4 primer (5′-TCCTCCGCTTATTGATATGC-3′), 0.025 U of DreamTaq DNA Polymerase (Thermo Fisher Scientific Baltics, UAB, Vilnius, Lituhania), and 10 µL of fungal template DNA (10–20 ng/μL). The PCR was performed in a MultiGene thermal cycler (Labnet International, Inc., Cambridge, UK). The PCR program included initial denaturation at 94 °C for 2 min followed by 34 cycles (94 °C for 1 min, 55 °C for 1 min, 72 °C for 2 min), and a final extension at 72 °C for 7 min. PCR products were detected by electrophoresis on 2% agarose gel dissolved in TBE 1× running buffer (Tris-borate-EDTA) (VWR International, Vienna, Austria), into which was added 0.5 µg/mL ethidium bromide, and visualized using a GelDoc-It Imaging System (Analytik Jena, Upland, CA, USA). Sequencing was performed at the Cellular and Molecular Immunological Application (CEMIA, Larissa, Greece). The generated sequences were then compared with the available sequences in the NCBI database (National Center for Biotechnology Information) using the BLASTN tool (https://blast.ncbi.nlm.nih.gov/Blast.cgi/) (accessed on 8 December 2021) to identify at specie level based on percent identity. 

### 2.3. Testing the Influence of Temperature, pH, and Sodium Chloride (NaCl) on Yeast Growth

All tests were performed in sterile tubes containing 10 mL of YEPD broth. Yeast strains were grown in YEPD broth for 24 h (around 10^7^ cells/mL). The cells were harvested by centrifugation at 4000× *g* for 5 min at 4 °C and resuspended in 0.9% sterile saline solution. The tested temperatures were 14 °C, 20 °C, and 26 °C. The pH was adjusted to the following different levels: 1.5, 2.5, 3.5, 4.5, 5.5, 6.5, and 7.5. The sodium chloride was tested at different concentrations between 0–3% (*w*/*v*). The inoculated tubes thus prepared were incubated at 26 °C for the pH and NaCl tests. Yeast growth was evaluated after 24 h of incubation by CFU/mL.

### 2.4. Auto-Aggregation and Hydrophobicity

Auto-aggregation and hydrophobicity activities were assessed using the methods described by Alkalbani et al. [57] with some modifications. Yeast strains were grown in YEPD broth for 48 h. The cells were harvested by centrifugation at 4000× *g* for 5 min at 4 °C and then washed twice with PBS 1× solution (phosphate buffered saline, pH 7.2, VWR International, Vienna, Austria) and adjusted to OD600 at 0.6 ± 0.05 (*A*0). Then, the absorbance (*At*) was measured at time intervals of 0, 2, 4, and 24 h at the OD600. The auto-aggregation activity was calculated using the following Equation (1):

(1) $$\mathrm{Auto}-\mathrm{aggregation}\%=\left(1-\frac{\mathrm{At}}{A0}\right)\times 100$$

The affinity of yeast isolates to hydrocarbons was determined using two solvents, hexane (VWR International, Rosny-sous-Bois, France) and xylene (VWR International, Rosny-sous-Bois, France). Cell suspension (3mL) in PBS (pH 7.2) with 1 mL of solvent were mixed for 2 min. After 1 h incubation at room temperature, the aqueous phase was carefully recovered and the absorbance was measured at OD_600_ (*A*1). The hydrophobicity percentage was expressed according to the Equation (2):

(2) $$Hydrophobicity\;\%=\left(\frac{A0-\;A1}{A0}\right)\times 100$$

### 2.5. Antioxidant Activity

Antioxidant activity was calculated according to the method reported by Brand-Williams et al. [59] with some modifications. Briefly, the fresh cell suspension was mixed 1:1 with the DPPH (1,1-Diphenyl-2-Picrylhydrazyl) dissolved in 100 µM methanol solution. The suspension was vortexed vigorously for 2 min and then stored at ambient temperature for 30 min in darkness. The samples were centrifuged at 2000× *g* for 5 min and the absorbance was measured at 517 nm. The blank solution was prepared using deionized water. The radical scavenging activity was expressed according to the Equation (3):

(3) $$\mathrm{Scavenging}\;\mathrm{rate}\;\left(\%\right)=\left(\frac{A\;\mathrm{DPPH}-\mathrm{Asample}}{\mathrm{ADPPH}}\right)\times 100$$

### 2.6. Yeast Survival Rate to Gastrointestinal Barriers In Vitro

Yeast survival rate to simulated gastrointestinal barriers was assessed using the method described by Diguță et al. [26] with slight modifications. Overnight cell cultures were centrifuged at 2000× *g*, for 10 min, washed twice, and resuspended with sterile PBS 1× solution. Simulated artificial gastric juice was prepared by suspending pepsin (0.3% *w*/*v*) in sterile buffer (PBS 1×) with pH adjusted to 2.0. An aliquot (around 10^7^ CFU/mL) of isolate was inoculated into 10 mL of simulated artificial gastric juice and then incubated at 26 °C in aerobic static conditions. Over time, an aliquot was taken at 0 min, 1 h 30 min, 3 h, and 24 h and diluted in PBS solution to evaluate cell viability by pour plating in YEPD Agar. The survival rate was defined as Equation (4):

(4) $$\%\;\mathrm{viability}=\left(\frac{\log\;\mathrm{CFU}\;\mathrm{Nt}\;}{\log\;\mathrm{CFU}\;\mathrm{Ni}}\right)\times 100$$

where Ni and Nt denote the log CFU.mL^−1^ at 0 time and after different time intervals, respectively.

Tolerance to bile salts was also assessed *in vitro*. An aliquot of the overnight cell cultures (around 10^6^ CFU/mL) of each strain was inoculated into 10 mL of YEPD broth (pH 8.0) supplemented with 0.3% bile salts, then incubated at 26 °C in aerobic static conditions for 4 h. Over time, an aliquot was taken at 0, 2, and 4 h intervals and diluted in PBS solution to evaluate cell viability by pour plating in YEPD Agar.

### 2.7. Antibacterial Activity

Antibacterial activity was evaluated against nine indicator pathogenic bacteria including *Bacillus cereus* ATCC 11778, *Escherichia coli* ATCC 25922, *Enterococcus faecalis* ATCC 29212, *Listeria ivanovii* ATCC 19119, *Listeria monocytogenes* ATCC 7644, *Staphylococcus aureus* ATCC 6538 MSSA (methicillin sensible), *Staphylococcus aureus* ATCC 43300 MRSA (methicillin-resistant), *Pseudomonas aeruginosa* ATCC 9027, and *Proteus vulgaris* ATCC 13315 by the cross-streaking method [60]. All reference bacteria were provided by the American Type Culture Collection (ATCC), (Manassas, VA, USA). Briefly, overnight yeast cultures were inoculated by a single streak in the center of the surface of YEPD Agar plates. After incubation at 26 °C for 48 h, the pathogenic strains were inoculated in a streak perpendicular to the yeast strains and incubated again for 24 h at 30 °C. Antibacterial activity was defined as a clear inhibitory zone formed and measured in millimeters (mm).

### 2.8. Antibiotic Susceptibility

The antibiotic susceptibility of the yeast strains was evaluated using the Kirby Bauer method, according to CLSI [61]. The following thirteen different antibiotics (BioAnalyse, Ankara, Turkey) were used: Ampicillin (AM-10), Cephalexin (CL-30), Chloramphenicol (C-30), Erythromycin (E-10), Lincomycin (L-10), Nalidixic Acid (NA-30), Vancomycin (VA-10), Clotrimazole (CTM-10), Fluconazole (FLU-10), Itraconazole (ITR-10), Ketoconazole (KCA-10), Miconazole (MCL-10), and Nystatin (NS-100). Briefly, an aliquot (100 μL) of each overnight yeast culture was spread onto YEPD Agar plates and then the antibiotic discs were placed on the surface of agar plates. After the incubation period, the diameters of inhibition zones around the discs were measured using a ruler and the results were expressed as sensitive (>15 mm) or resistant (≤15 mm) according to CLSI [61].

### 2.9. Catalase and Hemolytic Activity

A drop of the overnight yeast culture was mixed with a drop of 3% hydrogen peroxide on a microscope slide. The appearance of bubbles indicates a positive reaction.

Fresh yeast cultures (10 μL) were spotted on the surface of Columbia Agar with Sheep Blood Plus plates (Oxoid, Hampshire, UK) and incubated at 26 °C, for 48 h. *Staphylococcus aureus* ATCC 43300 was used as a positive control for β-hemolysis. Results are expressed as beta (β) hemolysis (complete lysis of the red blood cells which appears as a clear zone around colonies), alfa (α) hemolysis (partial hemolysis which appears as greenish zone around the colonies), and gamma (γ) hemolysis (no hemolysis). Strains that did not produce any change in the medium (γ-hemolysis) were considered safe.

### 2.10. Conditioning of Yeast by Freeze-Drying Procedure

Yeast conditioning by a freeze-drying procedure was carried out according to the method described by Diguță et al. [26] with slight modifications. The following three different cryoprotective agents were tested during the lyophilization procedure: glucose, maltodextrin, and sucrose having a final concentration of 5% (*w*/*v*). Cryoprotectant solutions were prepared by dissolving in distilled water and then sterilized by filtration with Millipore filters with a pore size of 0.22 μm (Merck, Darmstadt, Germany). Distilled water was used as a control. 

The yeast cells (stationary phase) were recovered by centrifugation (4000× *g* for 5 min). The cell pellet was washed twice with PBS 1× buffer (pH 6.5), and resuspended into the protective solution. After being frozen overnight at −20 °C, samples were desiccated in a chamber type freeze-dryer (FreeZone6, LABCONCO, 6 Liter Benchtop Freeze Dry System, Kansas, MO, USA) for 16 h at −55 °C and 0.3 mbar. Freeze-dried yeast strains were rehydrated with PBS 1× buffer. Viability was measured before and after freeze-drying using the plate counting method. Cell viability was expressed as a percentage using the following Equation (5):

(5) $$\%\;\mathrm{viability}=\left(\frac{\log\;\mathrm{CFU}\;\mathrm{Nt}\;}{\log\;\mathrm{CFU}\;\mathrm{Ni}}\right)\times 100$$

Ni and Nt denote the log CFU.mL^−1^ after and before freeze-drying, respectively.

### 2.11. Statistical Analysis

All of the experiments were performed in triplicate. The results were expressed as the mean ± standard deviation (SD). The CFU/mL results were converted to log_10_ CFU/mL. Data analysis was conducted using one-way analysis of variance (ANOVA), followed by the Tukey B test. *p* values below 0.05 were deemed statistically significant. Statistical analysis was carried out using IBM SPSS Statistics for Windows version 28 (IBM Corp., Armonk, NY, USA).

## 3. Results

### 3.1. Molecular Identification of Yeast Strains

The yeast strains were identified by 5.8S-ITS region sequencing at the specie level. BLASTN analysis was used for a homology search with different 5.8S-ITS sequences deposited in the NCBI database. The 5.8S-ITS region revealed that the strain BB06 showed 100% identity to *Saccharomyces cerevisiae* (*S. cerevisiae*), strain OBT05 was found to have 98.67% identity with *Metschnikowia pulcherrima* (*M. pulcherrima*), and MT07 showed 100% identity to *Torulaspora delbrueckii* (*T. delbrueckii*), respectively. 

The sequences were submitted to the NCBI database under the accession numbers listed in parentheses: *Metschnikowia pulcherrima* OBT05 (OL757481), *Torulaspora delbrueckii* MT07 (OL757482), and *Saccharomyces cerevisiae* BB06 (OL757483). 

### 3.2. Influence of Temperature, pH, and Sodium Chloride (NaCl) on Yeast Growth

**Figure 1:**
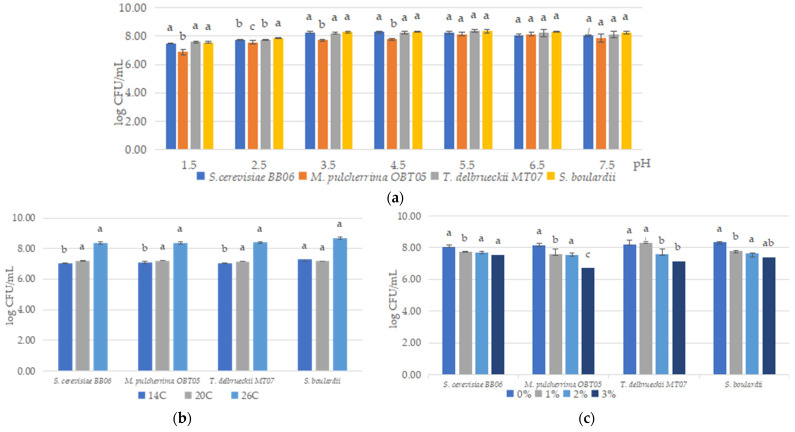
The ability of yeast strains to grow at different abiotic conditions: pH range (**a**), temperature (**b**), and NaCl concentration (**c**). Significant statistical differences (*p* < 0.05) between yeast strains according to the Tukey B test from one-way analysis of variance (ANOVA) have been indicated by different letters.

Different pH values (1.5, 2.5, 3.5, 4.5, 5.5, 6.5, and 7.5) were investigated to assess the capacity of yeast strains to survive at wide pH ranges. In our study, all yeast strains exhibited high tolerance to the lower pH levels, from 1.5 to 3.5, and maintained optimal growth cells at a pH of 4.5–7.5 (Figure 1a). As regards the tolerance to temperature, the experiments were carried out at 14, 20, and 26 °C, the typical temperatures of fishery water. Yeasts showed a high trend toward growth at lower temperatures (Figure 1b), with an optimum at 26 °C. Furthermore, all the yeast isolates had a high tolerance to NaCl, from 1.0 to 3.0% (*w*/*v*) (Figure 1c).

### 3.3. Auto-Aggregation and Hydrophobicity Ability

Auto-aggregation and cell hydrophobicity percentages of yeast strains are presented in Table 1. All yeast strains showed auto-aggregation (%) ranging between 45.70 ± 1.59% and 60.99 ± 2.77% after 2 h at 26 °C but, by extending the incubation time, this increased significantly, ranging between 73.44 ± 1.58% and 92.08 ± 1.49% after 24 h at 26 °C. The strongest auto-aggregation ability, after 24 h, was recorded for *S. cerevisiae* BB06 (92.08 ± 1.49%), which is significantly higher than the reference *S. boulardii.* A lower auto-aggregation percentage was observed for *M. pulcherrima* OBT05 (73.44 ± 1.58%) and *T. delbrueckii* MT07 (74.14 ± 0.27%) after 24 h.

Yeast strains revealed low to moderate hydrophobicity in hexane, which ranged between 5.93 ± 1.54% and 53.43 ± 1.09%, and in xylene, ranging between 19.03 ± 3.87% and 38.08 ± 0.90%, respectively. *S. cerevisiae* BB06 showed the highest degree of hydrophobicity toward hexane (53.43 ± 1.09%) compared to reference *S. boulardii* (32.84 ± 3.27%), and significantly higher than that of the other two yeast strains. Compared with the results reported previously, the hydrophobicity percentage in xylene for *S. cerevisiae* BB06 was lower (24.36 ± 0.36%), increasing for *T. delbrueckii* MT07 (19.03 ± 3.87%).

### 3.4. Antioxidant Properties

The antioxidant activities of yeast cellular suspensions were measured by DPPH assay. The DPPH-free radical-scavenging rate of *T. delbrueckii* MT07 reached 60.61 ± 1.32%, which was similar to reference *S. boulardii* (60.46 ± 0.80%) and significantly higher than that of the other two strains (Table 1). Moreover, all yeast strains had strong antioxidant properties (>55%) (Table 1).

### 3.5. Resistance to Gastric Acidity and Bile Salts

**Figure 2:**
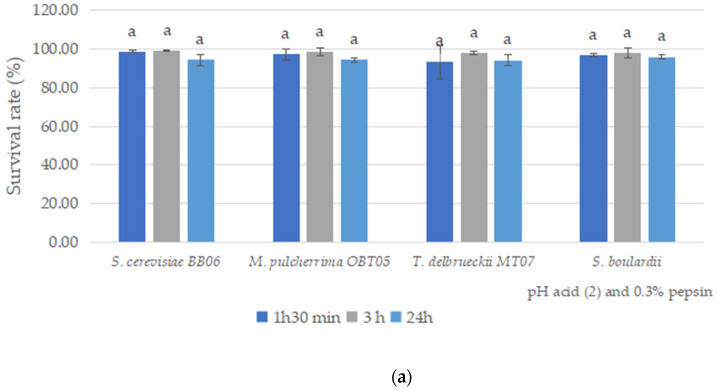

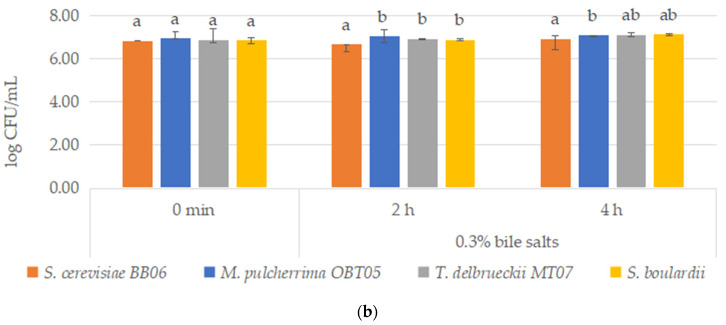
*In vitro* screening of probiotic properties of yeast strains under pH 2.0 and 0.3% pepsin (**a**) and 0.3% bile salts (**b**). Results are represented as mean ± SDs of three independent experiments. Significant statistical differences (*p* < 0.05) between yeast strains according to the Tukey B test from one-way analysis of variance (ANOVA) have been indicated by different letters.

The yeast strains were further examined to characterize their survival rates under simulated gastric conditions (pH 2.0 and 0.3% pepsin) (Figure 2a) and growth rates under intestinal fluids (bile salts at 0.3%) (Figure 2b). All yeast strains started the simulation with an average of 7 log CFU/mL.

After 90 min of incubation in artificial gastric juice, the four yeast strains showed a high survival rate of between 93.30 and 98.71%; *S. cerevisiae* BB06 was the most resistant, with a survival rate of 98.71% (Figure 2a). After 3 h of incubation, all yeast strains showed an improved tolerance, ranging between 98.01 and 99.32% (Figure 2a). The BB06 strain again exhibited a high rate of 99.32%. However, after 24 h of exposure under simulated gastric conditions, the yeast strains showed a slightly decreased rate of 94.25–95.96%. *S. boulardii* showed a better survival rate of 95.96% (Figure 2a). As regards the tolerance to intestinal conditions, a slight increase of viable cell counts was observed, with 0.09–0.30 log after 4 h of exposure to simulation conditions, which highlighted the high tolerance of all yeast strains at 0.3% bile salts (Figure 2b).

### 3.6. Antibacterial Properties

**Figure 3:**
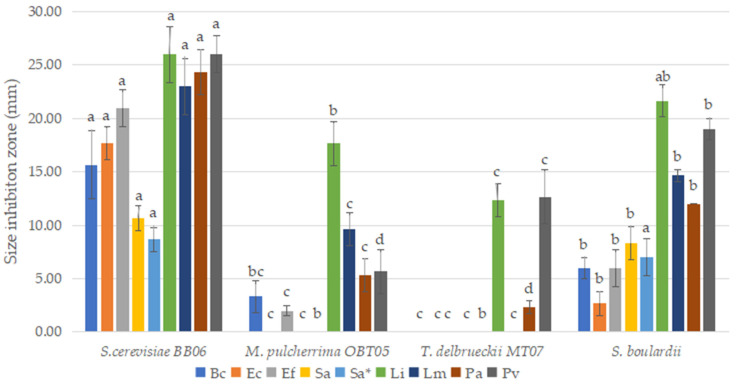
*In vitro* testing of the antibacterial activity of the yeast strains used against *B. cereus* ATCC 11778 (**Bc**), *E. coli* ATCC 25922 (**Ec**), *Ent. faecalis* ATCC 29212 (**Ef**), *L. ivanovii* ATCC 19119 (**Li**), *L. monocytogenes* ATCC 7644 (**Lm**), *S. aureus* ATCC 6538 MSSA (methicillin sensible) (**Sa**), *S. aureus* ATCC 43300 MRSA (methicillin-resistant) (Sa*), *P. aeruginosa* ATCC 9027 (**Pa**), and *P. vulgaris* ATCC 13315 (**Pv**). Results are represented as mean ± SDs of three independent experiments. Significant statistical differences (*p* < 0.05) between yeast strains according to the Tukey B test from one-way analysis of variance (ANOVA) have been indicated by different letters.

The antibacterial properties of yeast strains were explored against the following nine common pathogenic bacteria of fish: Gram-positive (*Bacillus cereus*, *Enterococcus faecalis*, *Listeria ivanovii*, *Listeria monocytogenes*, MSSA, and MRSA *Staphylococcus aureus*), as well as Gram-negative (*Escherichia coli*, *Pseudomonas aeruginosa, Proteus vulgaris*). Among the yeast strains used, *S. cerevisiae* BB06 showed high antibacterial activity against all the reference pathogenic bacteria (Figure 3). *M. pulcherrima* OBT05 strain shows relatively low antagonistic activity against several pathogens, namely *Enterococcus faecalis*, *Bacillus cereus*, *Pseudomonas aeruginosa*, *Proteus vulgaris*, and *Listeria monocytogenes*, the greatest inhibitory effect being on *Listeria ivanovii*. The lowest antibacterial activity was presented by the strain *T. delbrueckii* MT07, and against only *Listeria ivanovii*, *Proteus vulgaris*, and *Pseudomonas aeruginosa*. *S. cerevisiae* BB06 presented a higher antagonistic activity than reference *S. boulardii*.

### 3.7. Antibiotic Susceptibility

The antibiotic susceptibility profile of yeast strains to 13 antibacterial and antifungal antibiotics was assessed (Table 2). We found that *S. cerevisiae* BB06, *T. delbrueckii* MT07, and *S. boulardii* showed resistance to all antibiotics used, except nystatin. The *M. pulcherrima* OBT05 strain showed resistance to antibacterial antibiotics and sensitivity to all antifungal antibiotics.

### 3.8. Catalase and Hemolysis Assay

Concerning catalase activity, all strains were catalase-positive, with *M. pulcherrima* OBT05 and *T. delbruecki* MT07 exhibiting the highest activity.

Only microbial strains with γ-hemolysis are considered safe, according to EFSA. In our study, all yeast strains showed γ-hemolysis (the greenish or clear area around colonies were not detected), thus strengthening their safety to be used as potential probiotics.

### 3.9. Conditioning by Lyophilization

**Figure 4:**
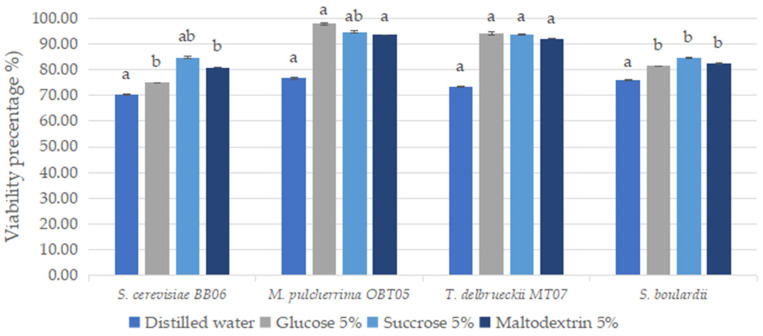
Conditioning by lyophilization of yeast strains with probiotic potential. Results are represented as mean ± SDs of three independent experiments. Significant statistical differences (*p* < 0.05) between yeast strains according to the Tukey B test from one-way analysis of variance (ANOVA) have been indicated by different letters.

Conditioning by lyophilization (or freeze-drying) of yeast strains, using three cryoprotective agents (glucose, maltodextrin, and sucrose in 5% concentration), resulted in obtaining a high percentage of viable yeast cells versus the control (no cryoprotectant), indicating better protection of the cells against stress factors during freeze-drying (Figure 4). High viability (>90%) of *M. pulcherrima* OBT05 and *T. delbruecki* MT07 strains was observed when the three cryoprotective agents were used (Figure 4). Among the protective substances used, sucrose improved the survival rate of *S. cerevisiae* BB06 and *S. boulardii* (average 85%) more than drying in a medium without cryoprotectants (between 70.25% and 75.87%). Moreover, including only water, the survival rate was higher than 70% for all yeast strains used (Figure 4).

## 4. Discussion

The biotechnological importance of yeasts is well known by their application as starter cultures in the production of high value functional food with health benefits [37,38,62]. Therefore, there is considerable interest in screening and characterization of new yeasts as potential probiotics. In this study, three yeast strains were isolated from agro-food natural sources (barley and grapes) and identified through the sequencing of the 5.8S-ITS region. Comparison with sequences deposited in the NCBI database revealed the presence of *Saccharomyces cerevisiae, Metschnikowia pulcherrima,* and *Torulaspora delbrueckii.* While *S. cerevisiae* and *S. boulardii* have been intensively studied due to their proven probiotic attributes (reviewed by Łukaszewicz [41]; Palma et al. [42]; Lazo-Vélez et al. [43]; Ansari et al. [44]), *M. pulcherrima* [47,63] and *T. delbrueckii* [47,48,50] have been less investigated. 

The next step was to assess *in vitro* the probiotic potential of the yeast strains based on the main selection criteria recommended in various review articles. 

We highlighted the ability of yeasts as probiotics to be effective in different environmental conditions such as wide pH ranges, temperature, and salinity variations. In our study, all yeast strains exhibited high tolerance to the lower pH, from 1.5–3.5, and maintained optimal cell growth at a pH of 4.5–7.5. Fishes are ectotherms, which means that their body temperature is influenced by the water temperature in which they are living, whether in the wild or captivity [64,65]. Water temperature can affect the functional activity of probiotics when they are administered as dietary supplements or in rearing water. In our study, it was found that yeast strains can grow at temperatures of 14–20 °C (average 7 log CFU/mL) with optimum growth at 26 °C (average 8 log CFU/mL). Hossain et al. [66] demonstrated the ability of yeast *S. boulardii* to grow at high temperatures of up to 50 °C, with optimum growth at 30 °C. Similar results have been obtained by Fakruddin et al. [67] and Andrade et al. [68]. Salinity stress in aquatic media can affects the survival rate of probiotic bacteria, which limits their application [14]. Yeasts possess certain advantages as compared to probiotic bacteria, such as salt tolerance. Our yeast strains were able to grow and tolerate salt concentrations (1–3%), which supports their use as probiotics in aquatic systems. Previously, Hossain et al. [66] demonstrated the capacity of yeast strains to grow at up to 8.0% NaCl concentration, with a rapid decrease of growth above 3.0% NaCl concentration. Similar results have been obtained by Fakruddin et al. [67] and Andrade et al. [68]. 

Other relevant probiotic traits have been studied, such as the ability of yeast strains to adhere to epithelial cells (auto-aggregation and hydrophobicity). Yeast strains showed a variable hydrophobic affinity in hexane and xylene and high auto-aggregation ability after extending the incubation time, which is in agreement with previously reported results [66,69].

In addition to functional properties studied for probiotic abilities, it is of interest for fish health to assess the antioxidant capacity. According to their percentage of antioxidant activity, yeast strains have been classified into the following five groups: very low (<20%), low (20–30%), good (30–40%), very good (40–50%), and excellent (>50%) [47]. Based on this classification, the yeasts studied in this work showed excellent antioxidant activity (>55%). Our results are strongly supported by previous studies. Romero-Luna et al. [69] reported that *S. cerevisiae* C41 had excellent antioxidant activity (63.03%), based on the reduction of DPPH radicals. Agarbati et al. [70] reported different *T. delbrueckii* strains possessing high antioxidant activity. However, Fernández-Pacheco et al. [50] reported two *Saccharomyces* strains with antioxidant capacity values of 33.71–32.66%, much lower than our *S. cerevisiae* BB06. In addition, all our yeast strains were positive for catalase. This activity contributes to the defense against reactive species [71]. 

One of the most desirable properties of probiotics is their antagonistic activity against pathogens. In several studies, the main bacterial pathogens have been identified that are transmitted from fish to humans when improperly cooked or raw fish or contaminated fish products are consumed [10,11]. In our study, *S. cerevisiae* BB06 showed a high broad spectrum of inhibition patterns against the reference Gram-positive and Gram-negative bacterial pathogens (against nine of nine pathogens), followed by *M. pulcherrima* OBT05 (against six of nine pathogens) and *T. delbrueckii* MT07 (against three of nine pathogens), respectively. *S. cerevisiae* BB06 presented high and more broad antagonistic activity than reference *S. boulardii*. Fadahunsi and Olubodun [63] reported that probiotic yeast *M*. *pulcherrima* showed antibacterial activity against food-borne pathogens such as *Campylobacter jejuni* and *Vibro cholerae*, and different *S. cerevisiae* strains against *Listeria monocytogenes*, *Campylobacter jejuni*, and *Salmonella* sp., respectively. Fakruddin et al. [67] reported that cell lysate of *Saccharomyces cerevisiae* IFST062013 showed better antibacterial activity than the whole cell and culture supernatant whole cells against Gram-positive bacteria (*B. subtilis*, *B. cereus*, *B. polymyxa*, *B. megaterium*, *E. faecalis*, and *S. aureus,)* and Gram-negative bacteria (*E. coli*, *K. pneumoniae*, *S. typhi*, *S. flexneri*, *P. vulgaris*, *P. aeruginosa* and *V. cholerae*). Agarbati et al. [70] reported yeast strains belonging to *Saccharomyces*, *Metschnikowia*, and *Torulaspora* with antimicrobial activity against *Candida albicans*, *E. coli*, *S. aureus*, and *Salmonella enterica*.

A key requirement for potential yeast probiotic strains is to survive in the gastrointestinal tract (GIT). The first challenge is the low acidity in the gastric conditions and our yeast strains successfully survived at pH 2.0 and the presence of 0.3% pepsin (survival rates ranging between 98.01 and 99.32%, after 3 h). We additionally demonstrated (as reported above) that our strains had good growth at 1.5 pH (Figure 1a). After this, bile tolerance is another essential criterion in the characterization of yeast strains as probiotics, because it could allow their growth in the intestinal tract. In our study, all yeast strains had a high ability to tolerate bile salts at 0.3%, after 4 h. Numerous studies have reported the capacity of probiotic yeast strains to tolerate up to 2% bile salts [72,73,74,75].

In addition to the studied probiotic properties of the yeast strains, the safety characteristics were evaluated. *S. cerevisiae* BB06, *T. delbrueckii* MT07, and *S. boulardii* showed resistance to antifungal antibiotics, except nystatin. *M. pulcherrima* OBT05 showed susceptibility to all antifungal antibiotics tested. In our study, all yeast strains showed resistance to antibacterial antibiotics, which makes them suitable for use during antibiotic treatment against pathogenic bacteria. No hemolytic activity was detected, which confirms the non-pathogenic character of the yeast strains, which can therefore be considered safe for use as probiotics in fish feed.

The preservation conditioning of probiotic yeasts is a relevant topic to their storage for long periods and maintaining probiotic traits. The most frequently drying methods such as spray-drying, freeze-drying (or lyophilization), vacuum-drying, and a fluidized bed with different upgrades have been used to encapsulate the probiotic preparations and deliver them in the form of dried biomass (reviewed by Frakolaki et al. [76] and Kiepś and Dembczyński [77]). Among these techniques, we chose freeze-drying for the preservation of yeast strains in a dry form. In our study, the addition of protective substances such as glucose, maltodextrin, and sucrose (in 5% concentration) protected *M. pulcherrima* OBT05 and *T. delbruecki* MT07 cells against stress factors during freeze-drying, and were found to be the best cryoprotectants, giving high survival rates (ranging from 91.99% to 97.85%). The survival rates of our yeast strains are much higher than those obtained by Nicolae et al. [78]. In this study, Nicolae et al. [78] reported cells survival rates of 35 and 77% for *S. cerevisiae*, *S. carlsbergensis*, and *Debaryomyces hansenii* mixed with sucrose 10% and gelatin 1.0% after freeze-drying. Arslan et al. [79] studied the effect of different wall materials including carbohydrates and proteins and two temperatures (80 °C and 125 °C) for spray-drying to microencapsulate yeast cells of *S. boulardii*. It was concluded that *S. boulardii* microencapsulated with gelatine and gum arabic displayed greater protection during spray-drying and simulated gastric conditions. Moreover, microencapsulated *S. boulardii* when spray-dried at 125 °C showed higher resistance under *in vitro* gastric conditions. 

The promising results obtained in this research study are encouraging, with reference to proceeding with *in vivo* trials to understand the mechanism of action of yeast strains as probiotics. Thus, the use of the *S. cerevisiae* BB06 strain should be established to determine the time required to colonize the gastrointestinal tract of fish and achieve the expected results. Finally, follow-up studies will focus on testing the functional and safety properties of S. *cerevisiae* BB06 to improve fish growth performance, feed digestion, disease control, or improving fish immunity, which will contribute to promote the use of probiotics in aquaculture.

## 5. Conclusions

Our results showed that the three yeast strains identified based on 5.8S-ITS region sequencing as *Saccharomyces cerevisiae*, *Metschnikowia pulcherrima*, and *Torulaspora delbrueckii* have valuable probiotic attributes in aquatic systems. *In vitro* examination of the influence of environmental conditions (temperature, pH, and salt stress) highlighted the ability of the yeast cells to survive under stressful conditions. High viability in the presence the 0.3% pepsin and low pH (2.0), and 0.3% bile salt, respectively, showed the strong abilities of yeast strains to survive passage through the fish gastrointestinal tract. The ability of yeast strains to respond to oxidative stress was highlighted by the positive results of the catalase test. Yeast isolates also displayed resistance patterns to antibacterial antibiotics and non-hemolytic activity. Overall, all yeast isolates had a strong antioxidant activity (>55%), high autoaggregation (between 73.44 ± 1.58% and 92.08 ± 1.49% after 24 h), and different cell surface hydrophobicity values with hexane and xylene (ranging from 5.93 ± 1.54% to 53.43 ± 1.09%). The obtained results of freeze-drying using sucrose as a cryoprotectant suggested that the yeast strains could be stored as the powdered formula. The BB06 strain showed the best combination of probiotic and healthy properties, which is why it is proposed to be used further for *in vivo* tests by including it in fish feed. 

## Figures and Tables

**Table 1:** Summarized characteristics and properties of yeast strains: auto-aggregation ability, hydrophobicity, and antioxidant activity.

Yeast Strains	Auto-Aggregation (%)			Hydrophobicity (%)		Antioxidant Activity (%)
	2 h	4 h	24 h	Hexane	Xylene	
*S. cerevisiae* BB06	60.51 ± 3.03 ^a^	81.01 ± 1.35 ^a^	92.08 ± 1.49 ^a^	53.43 ± 1.09 ^a^	24.36 ± 0.36 ^b^	55.97 ± 1.62 ^b^
*M. pulcherrima* OBT05	45.70 ± 1.59 ^b^	65.88 ± 1.43 ^b^	73.44 ± 1.58 ^b^	37.76 ± 1.08 ^b^	38.08 ± 0.90 ^a^	57.14 ± 2.85 ^ab^
*T. delbrueckii* MT07	47.24 ± 2.14 ^b^	64.68 ± 3.42 ^b^	74.14 ± 0.27 ^b^	5.93 ± 1.54 ^d^	19.03 ± 3.87 ^c^	60.61 ± 1.32 ^a^
*boulardii*	60.99 ± 2.77 ^a^	80.03 ± 0.67 ^a^	89.96 ± 1.55 ^a^	32.84 ± 3.27 ^c^	34.73 ± 0.99 ^a^	60.46 ± 0.80 ^a^

Results are represented as mean ± SDs of three independent experiments. Significant statistical differences (*p* < 0.05) between yeast strains according to the Tukey B test from one-way analysis of variance (ANOVA) have been indicated by different letters.

**Table 2:** Safety issues assessment of yeast strains.

Yeast Strains	Catalase Activity	Hemolytic Activity	Antibiotics Susceptibility												
			AM- 10	CL- 30	C- 30	E- 10	L- 10	NA-30	VA-10	CTM-10	FLU-10	ITR- 10	KCA- 10	MCL-10	NS- 100
*cerevisiae* BB06	+	Gamma	R	R	R	R	R	R	R	R	R	R	R	R	S
*pulcherrima* OBT05	++	Gamma	R	R	R	R	R	R	R	S	S	S	S	S	S
*delbrueckii* MT07	++	Gamma	R	R	R	R	R	R	R	R	R	R	R	R	S
*S. boulardii*	+	Gamma	R	R	R	R	R	R	R	R	R	R	R	R	S

Legend: +—positive reaction; Gamma (γ) hemolysis; R-resistant; S-sensitive; AM-10 (Ampicillin) CL-30 (Cephalexin), C-30 (Chloramphenicol), E-10 (Erythromycin), L-10 (Lincomycin), NA- 30 (Nalidixic Acid), VA-10 (Vancomycin), CTM-10 (Clotrimazole), FLU-10 (Fluconazole), ITR-10 (Itraconazole), KCA-10 (Ketoconazole), MCL-10 (Miconazole) and NS-100 (Nystatin).

## Data Availability

Not applicable.
